# The emerging role of lysine methyltransferase SETD8 in human diseases

**DOI:** 10.1186/s13148-016-0268-4

**Published:** 2016-09-22

**Authors:** Ciro Milite, Alessandra Feoli, Monica Viviano, Donatella Rescigno, Agostino Cianciulli, Amodio Luca Balzano, Antonello Mai, Sabrina Castellano, Gianluca Sbardella

**Affiliations:** 1 Dipartimento di Farmacia, Università degli Studi di Salerno, Via Giovanni Paolo II 132, Fisciano, I-84084 Salerno, Italy; 2 Epigenetic Med Chem Lab, Università degli Studi di Salerno, Via Giovanni Paolo II 132, Fisciano, I-84084 Salerno, Italy; 3 Programma di Dottorato di Ricerca in Scienze del Farmaco, Università degli studi di Salerno, Via Giovanni Paolo II 132, Fisciano, I-84084 Salerno, Italy; 4 Istituto Pasteur-Fondazione Cenci Bolognetti, Dipartimento di Chimica e Tecnologie del Farmaco, “Sapienza” Università di Roma, P.le A. Moro 5, I-00185 Rome, Italy; 5 Dipartimento di Medicina e Chirurgia, Università degli Studi di Salerno, Via Salvador Allende, Baronissi, I-84081 Salerno, Italy

## Abstract

SETD8/SET8/Pr-SET7/KMT5A is the only known lysine methyltransferase (KMT) that monomethylates lysine 20 of histone H4 (H4K20) in vivo. Lysine residues of non-histone proteins including proliferating cell nuclear antigen (PCNA) and p53 are also monomethylated. As a consequence, the methyltransferase activity of the enzyme is implicated in many essential cellular processes including DNA replication, DNA damage response, transcription modulation, and cell cycle regulation. This review aims to provide an overview of the roles of SETD8 in physiological and pathological pathways and to discuss the progress made to date in inhibiting the activity of SETD8 by small molecules, with an emphasis on their discovery, selectivity over other methyltransferases and cellular activity.

## Background

Lysine methylation on histone tails is a prevalent post-translational modification and, together with arginine methylation, plays a primary role in the regulation of chromatin structure and gene transcription, both in physiological and pathological conditions [1–5]. A large body of evidence indicates the importance of lysine methylation not only in epigenetic regulation of gene expression but also in the regulation of cellular signal transduction pathways [6, 7]. In fact, in addition to histones, a number of non-histone proteins are also methylated on Lys residues, leading to changes in their function or stability [7, 8]. Methylation increases the hydrophobic and basic nature of the lysine residue, which allows other proteins to recognize methylated lysine.

**Fig. 1:**
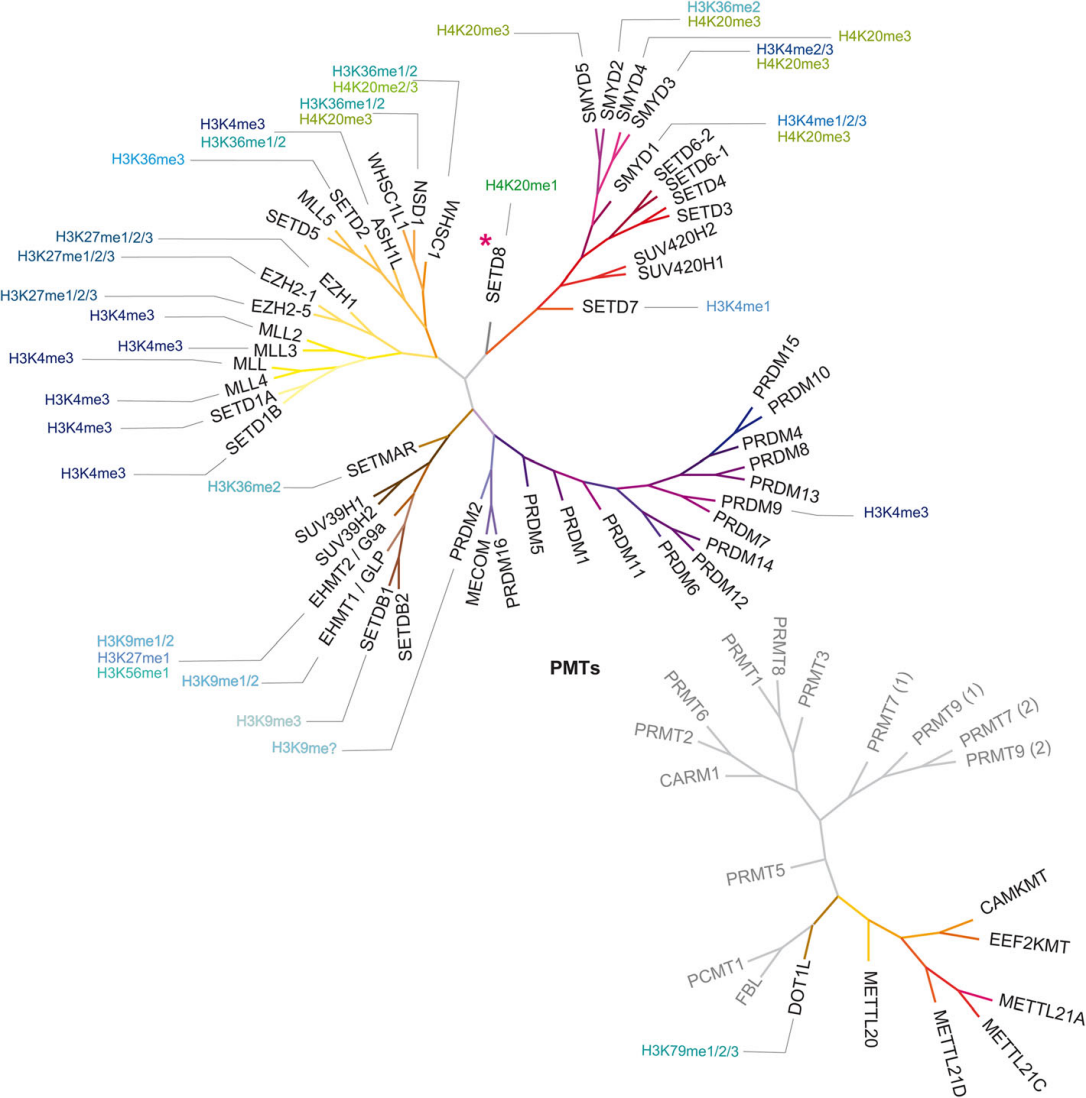
Phylogenetic tree of protein methyltransferases (PMTs; obtained with the Structural Genomic Consortium ChromoHub [102] and modified with Adobe Illustrator CS5). Specific modifications induced on histones H3 (shades of *blue*) and H4 (shades of *green*) are also shown. SETD8 is highlighted with a *rubine red asterisk*

There are three different forms of methylated lysines, monomethyl-, dimethyl-, and trimethyl-lysines [9], each one being produced by certain specific protein lysine methyltransferases (PKMTs or KMTs, Fig. 1) that catalyze the addition of a methyl group from *S*-adenosyl-*L*-methionine (SAM) to the ε-amine group of the side chain of a particular lysine residue [9]. Most KMTs contain the SET (Su(var), Enhancer of zeste, Trithorax) domain and are thought to catalyze a sequential bi-bi kinetic mechanism in which both substrate association and product release occur in a random manner [9]. Yet, numerous non-SET-domain proteins such as DOT1L and methyltransferase-like (METTL) proteins (Fig. 1) also have lysine N-methyltransferase activity [10, 11].

Many disease conditions, including cancer, have been associated with dysregulation of protein methylation and, indeed, there are a large number of reports describing that abnormal KMT activity drives cancer tumorigenesis or neurodegenerative diseases [12–15].

SETD8 (also known as PR-SET7, SET8, or KMT5A), a member of the SET domain containing family, is unique among the several KMTs identified so far because it is the sole mammalian enzyme known to catalyze the monomethylation of histone H4 Lys20 (H4K20me1, Fig. 1) [16, 17], a modification that may be involved in the recruitment of signaling proteins like 53BP1 to site of double-strand DNA breaks [18] or directly modulate chromatin compaction [19]. H4K20 methylation plays key roles in DNA replication, DNA damage repair, and silenced hetereochromatin [17, 20–22]. Consequently, there is a significant interest in the precise mechanistic role of this modification and the enzymes responsible for installing the methyl marks.

Here, we provide a summary of the most important activities of SETD8 in physiological and pathological pathways together with a comprehensive overview of SETD8 modulators (Tables 1 and 2).

**Table 1:** SETD8 roles in physiological and pathological pathways

Physiological/pathological pathways	Roles of SETD8	References
Cell cycle progression	A direct role in the regulation of ORIs	[44]
	Upregulated in G2/M and early G1, nearly absent in S phase	[17, 20, 35–40]
	SETD8 ubiquitin-mediated degradation is required for the onset of S phase	[34, 36–42]
Transcriptional regulation	Promotes transcriptional repression	[31, 42, 55–57]
	Mediates transcriptional activation	[20, 45, 52–54]
Regulation of DNA replication	Binds to the H4 N-terminal tail and blocks the acetylation of H4K5, H4K8 and H4K12 during G1, hindering DNA replication	[41]
DNA damage response	SETD8 methyltransferase activity during the DNA damage response is necessary to recruit 53BP1 for efficient DNA repair and checkpoint activation; enzyme depletion leads to an increase in spontaneous DNA damage	[18, 20, 49]
Regulation of p53 activity	Catalyzes p53 monomethylation (p53K382me1), suppressing p53-dependent transcription activation in cancer cells	[26]
	Methylates Numb (K158 and K163), thus uncoupling it from p53 and increasing p53 ubiquitination and degradation	[27]
Regulation of PCNA	Monomethylates PCNA (PCNAK248me1), thus stabilizing PCNA proteins through inhibition of polyubiquitylation and enhancing the interaction between PCNA and the flap endonuclease FEN1	[28]
Cancer	Overexpressed in different types of cancer tissues and cancer cell lines including bladder cancer, non-small cell and small cell lung carcinoma, chronic myelogenous leukemia, hepatocellular carcinoma, and pancreatic cancer	[28]
	SETD8-mediated p53K382me1 suppresses p53-dependent transcription activation in cancer cells	[26]
	SETD8-dependent monomethylation of PCNAK248 promote tumorigenesis	[28]
	Implicated in cancer invasiveness and metastasis through its interaction with TWIST	[60]
	Direct target of miRNA miR-127-3p, influencing OS progression and metastasis	[62]
	Reduced SETD8 expression by polymorphism rs16917496 T > C is associated with decreased susceptibility to different types of cancer (breast and ovarian cancer, SCLC, hepatocellular carcinoma, NSCLC, childhood ALL)	[63–68]
	miR-7-promoted SETD8 mRNA degradation inhibits H4K20 monomethylation and suppresses EMT and invasion of breast cancer cells	[61]
	Crucial for AR-mediated transcription activation of PSA gene	[69]
	SETD8 binding interaction is required for PRDM2 tumor suppressor function	[70]
Regulation of erythroid cells maturation	SETD8 is a repressor of endothelial transcription factor GATA-2 expression and regulates erythroid maturation and promotes the maturation and survival of definitive erythroblasts	[72–74]
Maintenance of adult skin	Loss of SETD8 results in loss of proliferation and impaired differentiation, accompanied by loss of the interfollicular epidermis and sebaceous glands	[75]
Regulation of adipogenesis	Upregulation of *Setd8* gene by PPARγ promotes adipogenesis, while gene knockdown suppresses it	[52]
Neurodevelopmental disorders	IUGR induces a reduction of PPARγ-SETD8-H4K20me1 and Wnt signaling, thus causing impaired neurodevelopment and subsequent neurocognitive impairment	[76]

**Table 2:** SETD8 inhibitors

compound	structure	Enzyme activity	Biological effects	Ref.
H acid		IC_50_ = 3.8 μM also inhibits EZH2 (3.0 μM); no inhibition of G9a, SETD7 and PRMT1	Not cell permeable	[79]
Thymolphthalein		IC_50_ = 9.0 μM also inhibits EZH2 (25.2 μM); no inhibition of G9a, SETD7 and PRMT1	Marked concentration-dependent effect on HeLa cells viability; evident reduction of H4K20me1; does not affect other histone methylation marks; possible interference with the binding to the nucleosome	[79]
EBI-099		IC_50_ = 4.7 μM no inhibition of G9a	Antiproliferative effects against human myelogenous leukemia K562 cells	[83]
MC1946 MC1948		IC_50_ = 3.3 μM and 2.6 μM, respectively; no inhibition of EZH2, G9a and SETD7	Both compounds (50 μM) reduce H4K20me1 in U937 cells; possible covalent inhibiton	[86]
MC1947 MC2569		Dual inhibitors of SETD8 (IC_50_ = 9.0 μM and 10.2 μM, respectively) and EZH2 (IC_50_ = 74.9 μM and 313.8 μM, respectively); no inhibition of G9a and SETD7	Both compounds (50 μM) reduce H4K20me1 in U937 cells; MC1947 induces massive cell death and increases granulocytic differentiation; possible covalent inhibition	[86]
Nahuoic acid A		IC_50_ = 6.5 μM no inhibition of G9a, EHMT1, SETD7, SUV39H2, SUV420H1, SUV420H2, DOT1L, PRMT3, PRMT5 and MLL complexes	SAM-competitive and substrate-noncompetitive; the compound inhibits SETD8 in U2OS osteosarcoma cells	[87, 88]
UNC0379		IC_50_ = 7.3 μM no inhibition of G9a, SETDB1, GLP, SUV39H2, SETD7, PRMT3, PRMT5-MEP50 complex, PRMT1, SUV420H1, SUV420H2, SMYD2, DNMT1, PRC2 complex, MLL1 complex, and DOT1L	Substrate-competitive and SAM-noncompetitive; no cellular activity reported	[95, 96]
SPS8I1 (NSC663284)		IC_50_ = 0.21 μM inhibits SETD2, G9a, SMYD2, CARM1, and PRMT3 with IC_50_ values in the low micromolar or submicromolar range; no inhibition of GLP, SETD7, PRMT1	Substrate-dependent and irreversible inhibitor of SETD8; reduces H4K20me1 in HEK293T cells and produces a cell cycle arrest phenotype; off-target inhibition of Cdc25	[97]
SPS8I2 (Ryuvidine)		IC_50_ = 0.50 μM inhibits GLP, SETD2, G9a, SMYD2, CARM1, and PRMT3 with IC_50_ values in the low micromolar or submicromolar range; no inhibition of SETD7, PRMT1	Substrate- and cofactor independent; irreversible inhibitor of SETD8; reduces H4K20me1 in HEK293T cells and produces a cell cycle arrest phenotype; off-target inhibition of cyclin-dependent kinase 4 and 2 (CDK4/2)	[97]
SPS8I3 (BVT948)		IC_50_ = 0.70 μM inhibits SETD2, G9a, SMYD2, CARM1, and PRMT3 with IC_50_ values in the low micromolar or submicromolar range; no inhibition ofGLP, SETD7, PRMT1	Substrate- and SAM-dependent; irreversible inhibitor of SETD8; reduces H4K20me1 in HEK293T cells and produces a cell cycle arrest phenotype; off-target inhibition of protein tyrosine phosphatase PTB1B	[97]
SGSS05-N SGSS05-NS SPECS21		Inhibit SETD8 with IC_50_ values ≤ 5 μM also inhibit SETD2, SETDB1, GLP, G9a, SMYD2, SMYD3, MLL1, SETD7, PRMT1, PRMT3, CARM1, PRMT8 in the low micromolar range		[98]

### SETD8 roles in physiology and pathology

#### SETD8 targets

**Fig. 2:**
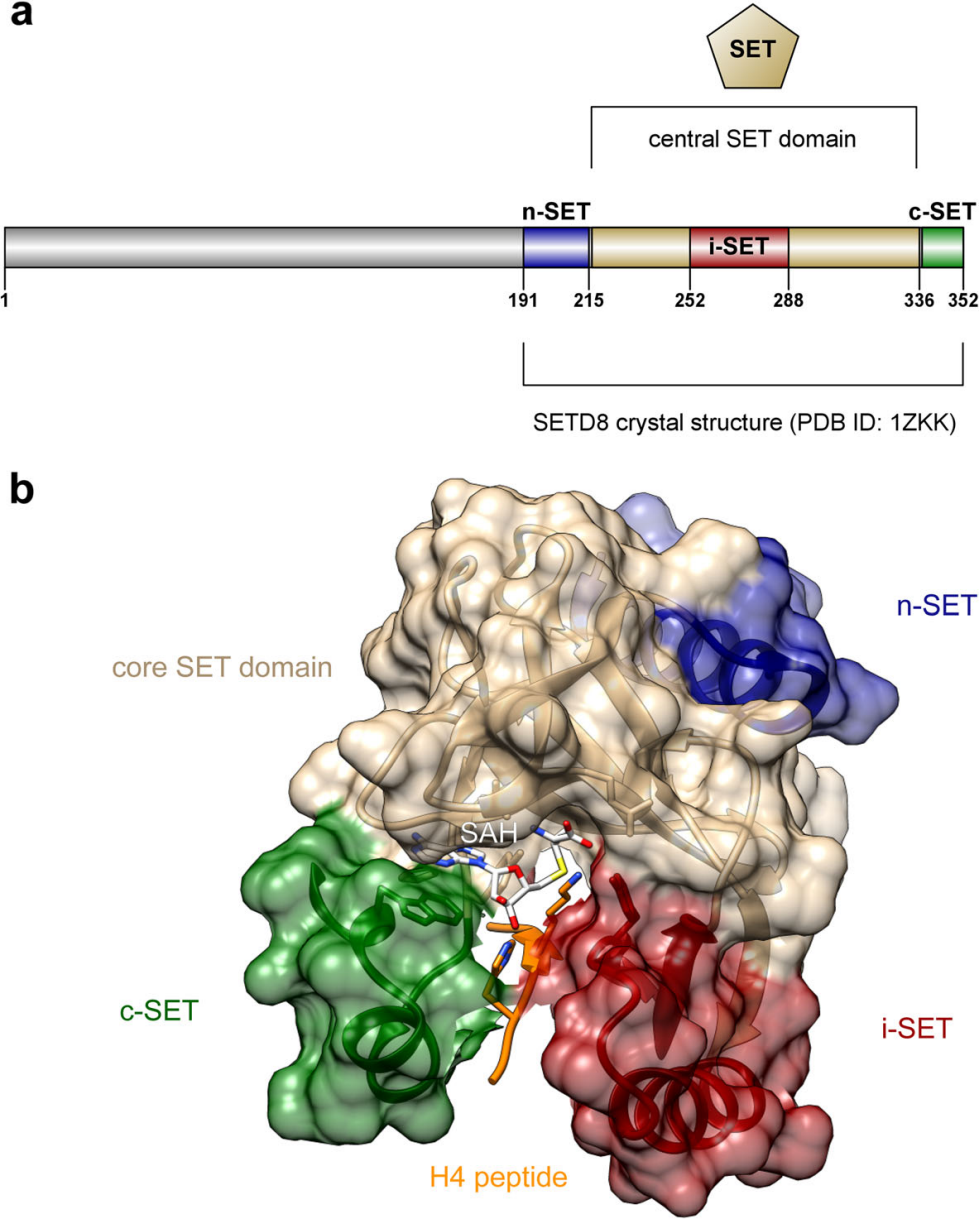
SETD8 protein structure. **a** Cartoon showing the C-terminal core SET domain and the locations of the n-SET, i-SET, and c-SET regions (obtained with). **b** Crystal structure of the SETD8 SET domain (*tan*), showing bound H4 peptide and substrate residue Lys20 (*orange*), n-SET (*blue*), i-SET (*dark red*), c-SET (*dark green*) regions, and product cofactor S-adenosyl homocysteine (SAH; *white*). The picture was prepared using Illustrator for Biological Sequences (IBS) [103] (panel **a**) and UCSF Chimera [104] (panel **b**, coordinates from Protein Data Bank (PDB) ID code *1ZKK*, chain A) and modified with Adobe Illustrator CS5

Differently from other KMTs, SETD8 prefers nucleosomes as substrates over histone proteins or peptides [16, 23], and this hints that the enzyme must interact with other surfaces of the nucleosome in addition to the H4 N-terminal region surrounding the targeted H4K20 residue. As a matter of fact, Tan and coworkers recently showed that the enzyme binds and methylates nucleosome substrates using multivalent interactions [24]. In fact, SETD8 uses at least three distinct regions to interact with the nucleosome substrate (Fig. 2). A basic N-terminal region is the primary determinant of such binding and interacts with a cluster of eight acidic residues of the nucleosomal H2A/H2B histone dimer (E56, E61, E64, D90, E91, E92 of H2A and E102, E110 of H2B) that forms a negatively charged “acidic patch” having the shape of a narrow groove [25]. The same basic N-terminal region of SETD8 likely interacts also with nucleosomal DNA (together with i-SET and c-SET domains) to anchor the methyltransferase to the nucleosome. This places the SET domain for interaction with the nucleosome face close to the targeted methylation site. Subsequently, the enzyme catalytic site engages the H4 tail and methylates lysine 20 [24].

**Fig. 3:**
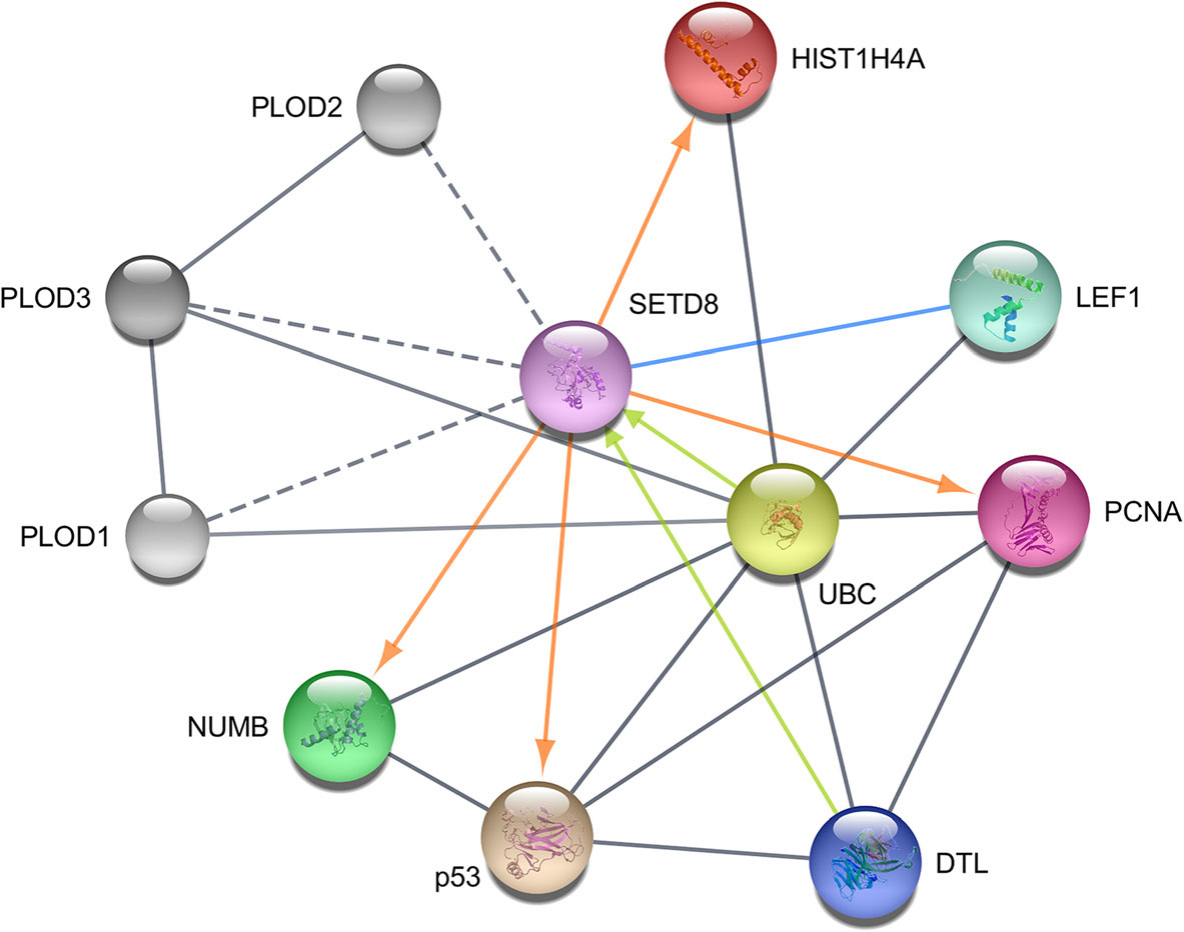
SETD8 protein–protein interaction network, obtained with Cytoscape v. 3.4.0 [105] using STRING database [106]. Only proteins directly interacting with SETD8 are displayed as colored glass-like marbles: histone cluster 1, H4a (HIST1H4a, *red*), lymphoid enhancer-binding factor 1 (LEF1, *aquamarine*), proliferating cell nuclear antigen (PCNA, *violet red*), denticleless protein homolog (DTL, also known as CDT2, *persian blue*), tumor protein p53 (p53, *tan*), protein numb homolog (NUMB, *green*). SETD8 is depicted in orchid. Direct interactions of SETD8 with protein targets leading to post-translational modifications are depicted as *orange arrows*, protein–protein interactions leading to SETD8 degradation as *lime arrows*, binding interactions as *cornflower lines*. Other interactions are shown as *gray lines*. Interactions of SETD8 with proteins PLOD1–3 (procollagen-lysine, 2-oxoglutarate 5-dioxygenase, *shades of gray*) were also retrieved from the interrogation of the STRING database, yet we found no evidence in the literature. Therefore they are shown as *dotted gray lines*

Besides H4K20, SETD8 also methylates lysine residues of many other proteins, including p53 and PCNA (Fig. 3). Gozani and coworkers reported that the enzyme monomethylates p53 at lysine 382 (p53K382me1) and that this methylation suppresses p53-dependent transcription activation in cancer cells [26]. The apoptotic pathway mediated by p53 is further regulated by SETD8 through the methylation of Numb, a protein existing in multiple isoforms in mammals and that was shown to form a tripartite complex with p53 and the E3 ubiquitin ligase MDM2 [27]. Numb promotes apoptosis in a p53-dependent manner, but the apoptotic function is abolished when Numb is methylated by SETD8 on the K158 and K163 residues contained in its phosphotyrosine binding (PTB) domain. Such methylation uncouples Numb from p53, resulting in increased p53 ubiquitination and degradation [27]. Also, Hamamoto and coworkers reported that SETD8 monomethylates lysine 248 of PCNA (PCNAK248me1), thus stabilizing PCNA proteins through inhibition of polyubiquitylation and substantially enhancing the interaction between PCNA and the flap endonuclease FEN1 [28].

#### SETD8 interactors and regulation

Within cells, SETD8 specifically localizes to mitotic chromosomes. In particular, it colocalizes with SIRT2 at mitotic foci. During mitosis, the association of the enzyme with chromosomes is increased in a H_2_O_2_-induced oxidative stress-dependent manner. Also, the protein associates with silent chromatin on euchromatic arms, whereas it does not associate with constitutive heterochromatin [29–33].

The regulation of H4K20me1 mediated by SETD8 during distinct cell cycle phases is essential for proper cell cycle progression as well as in the DNA damage response, and it was found associated with mitotic chromosomes during cell division. Recent data demonstrate a direct involvement of H4K20me1 modification in the assembling of the pre-replication complex (pre-RC) on the replication origins (ORIs) of metazoans [34]. Knockout of SETD8 and subsequent loss of H4K20me1 induce abnormalities in cell cycle progression and impaired nuclear condensation and are embryonic lethal preimplantation at approximately the 4- to 8-cell stages [35]. Also, SETD8 protein expression is tightly regulated during the cell cycle, being highest during G2/M (when it is targeted to ORIs to place H4K20me1) and early G1 and nearly absent during S phase [17, 20, 35–40]. During G1 and G2 phases, the enzyme is concentrated in the nucleus and is excluded from it during S phase. At G1/S transition, SETD8 is degraded by the proteasome through DCX^DTL^ (also known as CRL4^Cdt2^) and SCF^β-TRCP^ ubiquitin-mediated destruction, this degradation being required for the onset of S phase [34, 36–42]. (Wang) One of the two proliferating cell nuclear antigen (PCNA)-interacting domains carried by SETD8 is required for degradation of the enzyme [43]. Preventing SETD8 degradation in the S phase results in the accumulation of SETD8 at foci that co-localize with markers of active replicating forks (BrdU, PCNA, and DNA polymerase ε), further supporting a direct role for SETD8 in the regulation of ORIs [44].

Li and coworkers showed that SETD8 is directly involved into Wnt/β-catenin signaling, which plays an important role in a wide range of biological and pathophysiological processes [45]. They found that, under Wnt3a stimulation, the enzyme is recruited by lymphoid enhancing factor-1 (LEF1)/TCF4 to regulate the transcription of Wnt-activated genes, possibly through H4K20 monomethylation at the target gene promoters.

Chang and coworkers reported that SETD8 suppress DNA replication through histone binding. They showed that during G1, the enzyme binds to hypoacetylated but not hyperacetylated H4 N-terminal tail and blocks the acetylation of lysine residues K5, K8, and K12 of histone H4. Such a blockage can hinder DNA replication [41].

DNA damage response is a signaling pathway activated by DNA double-strand breaks which recruit signaling proteins to chromatin flanking the lesion via protein–protein interactions and post-translational modifications. H4K20 methylation is implicated in the DNA damage response in diverse organisms and in an evolutionarily conserved manner. In mammalian cells, depletion of SETD8 results in the formation of γH2AX foci (a hallmark of DNA damage) and increased sensitivity to a variety of genotoxic stresses, due to defects during DNA replication or mitosis and consequent genomic instability [20, 35, 46]. The recruitment of factors involved in the DNA damage response involves the interaction between histone modifications and specific reader domains, typically containing a hydrophobic pocket made up of two to four aromatic residues that can discriminate between the site and the degree of methylation by interacting with the methylated lysine as well as making additional contacts with the sequence surrounding the methylated residue [47, 48]. Both H4K20me1 and H4K20me2 are bound by a conserved tandem tudor domain contained in 53BP1, a key protein in DNA damage-signaling pathways that associates with sites of DNA damage at an early stage in the repair pathway [49]. Reinberg and coworkers showed that SETD8 is rapidly recruited to sites of DNA double-strand breaks through its binding to PCNA, inducing a prompt increase of H4K20me1 that is necessary to recruit 53BP1, functioning as a scaffolding protein for efficient DNA repair and checkpoint activation [20]. SETD8 methyltransferase activity during the DNA damage response is required for recruitment of 53BP1 [18].

Since it is enriched during mitosis, H4K20me1 represents a specific tag for epigenetic transcriptional regulation [16, 23, 30, 33, 50, 51]. However, regardless of numerous studies examining its role, this modification still has an uncertain function in gene expression, with evidence supporting a correlation with both gene expression and repression [20, 40]. Some gene-specific analyses, together with genome-wide ChIP studies, have associated H4K20me1 with active transcription [20, 45, 52–54], while a subset of non-genome-wide ChIP along with biochemical studies have correlated H4K20me1 with gene repression [31, 42, 55–57].

These apparently contradictory experimental results suggest that the impact of SETD8 on gene expression may be genomic- and/or cellular context-dependent. For instance, neighboring modifications on the H4 tail like as H4K16 acetylation (H4K16ac) may affect H4K20me1 function [20]. Recently, it has been shown that SETD8-mediated monomethylation of H4K20me regulates RNA polymerase II (Pol II) pausing dynamics and can function in both gene activation and repression [58]. In fact, H4K20me1 promotes the local recruitment of the MSL complex and acetylation of H4K16, thus inducing the release of RNA polymerase II (Pol II) into active elongation. In the absence of SETD8, MSL recruitment and H4K16Ac levels are locally down-regulated, Pol II pausing is induced, and gene expression is down-regulated. On the other hand, H4K20me1 serves as a substrate for SUV420H2-mediated H4K20me3 and imposes Pol II pausing. Down-regulation of SETD8 results in the local depletion of H4K20me2/3 at genes controlled by an H4K20me3-mediated pause but does not result in relief of paused Pol II because of an inability to recruit MSL in the absence of H4K20me1 [58].

#### SETD8 in pathology and physiology

As recently demonstrated by Hamamoto and coworkers, SETD8 is overexpressed in different types of cancer tissues and cancer cell lines including bladder cancer, non-small cell and small cell lung carcinoma, chronic myelogenous leukemia, hepatocellular carcinoma and pancreatic cancer [28]. They also reported that reduction or loss of SETD8-mediated methylation of PCNA significantly suppresses the growth of cancer cells by retarding the maturation of Okazaki fragments and slowing DNA replication, and demonstrated a correlation between the expression levels of SETD8 and PCNA in human cancer tissues [28]. Since PCNA has been widely recognized as a tumor marker for cancer progression and poor patient prognosis [59], SETD8-dependent PCNA methylation is likely to promote tumorigenesis. Similarly, SETD8 is overexpressed in different types of cancer tissues and cancer cell lines including bladder cancer, non-small cell and small cell lung carcinoma, chronic myelogenous leukemia, hepatocellular carcinoma and pancreatic cancer [28].

Moreover, SETD8 is implicated in cancer invasiveness and metastasis through its interaction with TWIST, a master regulator in epithelial–mesenchymal transition (EMT) [60]. It has been shown that SETD8 and TWIST are functionally interdependent in promoting EMT and enhancing the invasive potential of breast cancer cells *in vitro* and *in vivo*. Also, the KMT enzyme acts as a dual epigenetic modifier on the promoters of *E-cadherin* and *N-cadherin* the target genes of TWIST. Interestingly, the down-regulation of SETD8 by shRNA in breast cancer cells suppresses cell intravasation and spontaneous lung metastasis in an orthotopic mouse model [60]. Sun and coworkers reported that a microRNA (miRNA), miR-7, is a negative regulator of SETD8, inhibits H4K20 monomethylation, and suppresses EMT and the invasive potential of breast cancer cells [61]. MiRNAs are a class of small (∼20–22 nt) non-coding RNA molecules that regulate gene expression through binding the 3′-untranslated region (3′UTR) of targeted mRNA. The authors demonstrated that SETD8 is a downstream target of miR-7 that binds to SETD8 3′-UTR and inhibits the formation of H4K20me1 by promoting SETD8 mRNA degradation. This interaction suppresses EMT and invasion of breast cancer cells [61].

SETD8 has been also identified as a direct target of miR-127-3p, a miRNA that acts as a tumor suppressor in osteosarcoma (OS) tissues and cell lines and whose down-regulation in cancer may contribute to OS progression and metastasis. MiR-127-3p has been suggested to act mainly via the suppression of SETD8 expression. SETD8 overexpression can reverse the potential influence of miR-127-3p on the migration and invasion of OS cells [62]. A single-nucleotide polymorphism (SNP), namely polymorphism rs16917496 T>C, which is located within the binding site of another miRNA, miR-502, in SETD8 3′UTR, modulates SETD8 protein expression, and thus contributes to susceptibility to breast and ovarian cancer, and clinical outcome of small cell lung cancer and hepatocellular carcinoma [63–66]. It has been reported that the same SNP rs16917496 T>C contributes to the survival of non-small cell lung cancer (NSCLC) patients by altering SETD8 expression through modulating miRNA-target interaction [67] and is also associated with decreased risk of developing pediatric acute lymphoblastic leukemia (ALL) [68].

Ren, Sun, and coworkers demonstrated that SETD8 interacts with androgen receptor (AR) and that such interaction and H4K20me1 levels are crucial for AR-mediated transcription activation of PSA (prostate-specific antigen) gene [69].

Rice and coworkers reported a specific and direct binding of SETD8 with the Riz1/PRDM2/KMT8 tumor suppressor and showed that the N-terminal domain of PRDM2 preferentially monomethylates H3K9, thus establishing a H4K20me1-H3K9me1 trans-tail “histone code” [70, 71]. Both SETD8 binding domain and methyltransferase activity are essential for PRDM2 tumor suppressor function, and frameshift mutations resulting in a truncated protein incapable of binding SETD8 are a frequent hallmark of various aggressive cancers [70].

Besides its role in cancer diseases, it has been reported that SETD8 is also involved in other physiological and pathological processes, e.g. the regulation of erythroid maturation [72]. Steiner and coworkers recently reported that in erythroid cells, where expression levels of the enzyme are significantly higher than in any other cell or tissue type, SETD8 plays a crucial role as regulator of erythroid maturation, functioning primarily as a repressor of endothelial transcription factor GATA-2 expression. They showed that knockdown of SETD8 impair erythroid maturation and result in a delay in hemoglobin accumulation, larger mean cell area, persistent expression of mast/stem cell growth factor receptor CD117, incomplete nuclear condensation, and lower rates of enucleation, whereas cell proliferation or viability or DNA damage are not affected [72]. At the same time, following up their previous work establishing SETD8 as a context-dependent GATA-1 corepressor [73], Bresnick and coworkers demonstrated that SETD8 promotes the maturation and survival of definitive erythroblasts without involving upregulation of the established regulator of erythroblast survival Bcl-x_L_. They showed that SETD8-catalyzed H4K20me1 at a critical *Gata2* cis element restricts occupancy by Scl/TAL1, an enhancer of *Gata2* transcription, thus repressing *Gata2* transcription [74]. They also demonstrated that *Gata2* repression by SETD8 occurs in proerythroblasts, in which *Gata2* is not completely transcriptionally inactive, whereas in the more mature basophilic erythroblast the gene is silenced and insensitive to SETD8 downregulating effect. As a consequence, they postulated a mechanism in which the methyltransferase enzyme is required for initiation but not maintenance of *Gata2* repression [74].

Frye and coworkers reported that SETD8 is also necessary for the maintenance of adult skin and is required for c-Myc-induced epidermal proliferation [75]. In the epidermis, during the development as well as in adult skin, loss of SETD8 results in loss of proliferation and impaired differentiation, accompanied by loss of the interfollicular epidermis and sebaceous glands. The loss of differentiation is a result of the lack of H4K20me1-mediated activation of p63 gene, which is expressed in basal cells and is thought to function as a master regulator of the stratification of the developing epidermis. On the other hand, the failure of *Setd8*-null skin to proliferate is mostly due to increased expression of p53, which results in increased apoptosis in the basal layer of the epidermis [75].

Other studies showed that adipogenesis is also epigenetically regulated through H4K20 monomethylation through a feedback loop [52]. In fact, Sakai and coworkers reported that *Setd8* gene is upregulated by peroxisome proliferator-activated receptor γ (PPARγ) and that the knockdown of the gene suppresses adipogenesis. Interestingly, they showed that toward the end of differentiation SETD8-catalyzed H4K20me1 levels are markedly increased and positively regulate the expression of PPARγ and its targets [52]. Furthermore, the activation of PPARγ transcriptional activity leads to the induction of H4K20me1 modification of the receptor protein and its targets and thereby promotes adipogenesis. They also reported that H4K20me1 is involved into the PPARγ2 targeting by PPARγ promoting its gene expression [52].

PPARγ pathway is also important in the intrauterine growth restriction (IUGR) that occurs when a fetus fails to reach optimal growth potential in utero, frequently as a result of maternal hypertensive disorders and uteroplacental insufficiency. IUGR and other insults occurring during developmentally plastic periods cause neurodevelopmental impairment and long-term neurological morbidities that are evident as early as 2 years of age and persist beyond school entry. In IUGR infants long-term neurological morbidities are correlated with changes in brain connectivity and reduced volume of the hippocampus, a brain region key for the formation of certain types of memory. Joss-Moore and coworkers demonstrated that IUGR induces a reduction of the levels of PPARγ, SETD8 and H4K20me1 in juvenile rat hippocampus in conjunction with reduced Wnt signaling [76]. Wnt3a, one of the Wnt signaling genes, is crucial for normal growth of the hippocampus and regulate the expansion of the caudomedial cortex, from which the hippocampus develops. Moreover, Axin2, another Wnt signaling target gene, is essential for myelination and remyelination in brain development. Since canonical Wnt/β-catenin signaling is activated by SETD8- catalyzed H4k20me1 which is decreased in hippocampus, the authors hypothesized that reduced PPARγ-SETD8-H4K20me1 and Wnt signaling may contribute to altered hippocampal cellular composition which, in turn, may contribute to impaired neurodevelopment and subsequent neurocognitive impairment in IUGR offspring [76].

### SETD8 inhibitors

Despite the importance of selective SETD8 inhibitors as chemical probes to further investigate the cellular effects of SETD8 inhibition in both normal and diseased cells and as lead structures for the development of novel therapeutics, only a limited number of such compounds have been reported so far. Moreover, just a few of them are endowed with a certain degree of selectivity.

The first reported inhibitors of SETD8 were two dye-like compounds, H acid and thymolphthalein [77, 78], described by Reinberg et al. in a 2007 patent [79]. The two compounds were identified from the screening of a small focused library [80–82] against three SET-containing KMTs, SETD8, H3K9-specific G9a, and H3K4-specific SETD7, as well as the H4R3-specific arginine methyltransferase PRMT1. Both of them showed low IC_50_ values against nucleosomal KMTs SETD8 (3.8 and 9.0 μM, respectively) and EZH2 (3.0 and 25.2 μM, respectively), while were inactive against the other tested enzymes [79]. H acid is unable to enter cells, whereas thymolphthalein induces a marked and concentration-dependent reduction of HeLa cells viability and an evident reduction of global H4K20me1. Other histone methylation marks are not affected. Also, the compound induces a significant time- and concentration-dependent enrichment of the mitotic population and abolishes the DNA stimulatory effect on SETD8 methyltransferase activity when octamers or H4-peptides are used as substrates [79]. Thymolphthalein might possibly generate a conformational change within the SET domain of the enzyme that impairs catalysis or, otherwise, interfere with the binding of the enzyme with the nucleosome structure [24].

A few years later, Kodama et al. reported in another patent the identification of a few compounds, namely derivatives EBI-099, EBI-435 and EBI-455, from the virtual screening of about 2 million compounds [83]. Among them, EBI-099 inhibits SETD8 (IC_50_ = 4.7 μM) but not G9a and shows antiproliferative effects against human myelogenous leukemia K562 cells [83].

In the context of a series of studies aimed at the identification of epigenetic multiple ligands targeting different enzymes [84, 85], in 2012, Mai and coworkers identified a few bis(bromo- and dibromo-methoxylphenol) derivatives as inhibitors of SETD8. Among them, derivatives MC1946 and MC1948 (indicated as compounds 5 and 10, respectively, in the paper) are selective against SETD8, whereas bis(monobromo) analogs MC1947 and MC2569 (indicated as compounds 4 and 9, respectively, in the paper) show dual SETD8 and EZH2 inhibition. The compounds also reduce H4K20me1 levels in U937 cells, after 24-h treatment at 50 μM. Compound MC1947 also induces massive cell death and increases granulocytic differentiation [86]. Since they contain an electrophilic α,β-unsaturated carbonylic system, all MC derivatives could inhibit SETD8 via a covalent binding.

In 2013, Andersen and coworkers isolated the polyketide nahuoic acid A from cultures of a *Streptomyces sp.* obtained from a marine sediment and reported its inhibitory activity against SETD8 (IC_50_ = 6.5 ± 0.5 μM) without any significant effect on the activity of other protein methyltransferases such as G9a, EHMT1, SETD7, SUV39H2, SUV420H1, SUV420H2, DOT1L, PRMT3, and PRMT5 and MLL complexes [87]. Nahuoic acid A competes with SAM binding (*K*_*i*_ = 2 ± 0.3 μM) and is noncompetitive with respect to the binding of the peptide substrate. Even if the structure of the compound is rather polar, the authors have recently reported that both nahuoic acid A and its pentaacetate analogue are able to inhibit proliferation of several cancer cell lines in vitro with modest potencies [88]. The compound also appeared to selectively inhibit SETD8 in U2OS osteosarcoma cells. A few analogs, nahuoic acids B–E [89], differing from nahuoic acid A in the hydroxylation patterns on the decalin ring system and in the length and functionality of the C-13 side chain, show similar inhibiting effects on SETD8 activity [88].

In 2014 Jin and coworkers identified compound UNC0379 from the cross-screening against SETD8 of their library of >150 quinazoline-based compounds targeting lysine methyltransferases or methyllysine reader proteins [90–94]. The compound inhibits SETD8 (IC_50_ = 7.3 ± 1.0 μM) and is competitive with the peptide substrate and noncompetitive with the cofactor. Moreover, it has been reported as selective for SETD8 over 15 other methyltransferases [95]. The authors also studied the structure-activity relationships of UNC0379 and a few analogs [96]. However, cellular activities have not been reported.

Almost at the same time, Luo and coworkers identified three SETD8 inhibitors from the screening of a library consisting of more than 5000 commercially available compounds [97]. The three compounds SPS8I1–3 (also known as NSC663284, BVT948, and ryuvidine, respectively) are potent inhibitors of SETD8 (apparent IC_50_ values of 0.21 ± 0.03, 0.50 ± 0.20, and 0.70 ± 0.20 μM, respectively) and are not active against GLP, SETD7, and PRMT1, with the exception of SPS8I2 that inhibits GLP with IC_50_ of 4.70 ± 0.30 μM. Yet, the three compounds inhibit SETD2, G9a, SMYD2, CARM1, and PRMT3 with IC_50_ values in the low micromolar or submicromolar range. Mechanistic studies suggested different mode of action for the inhibition of SETD8 by SPS8I1 (substrate dependent), SPS8I2 (no substrate or SAM dependence), and SPS8I3 (both substrate and SAM dependent) and showed that SPS8I1–3 inhibits SETD8 via an irreversible slow-onset process involving cysteine residues [97].

In human embryonic kidney HEK293T cells, the compounds reduce H4K20me1 whereas other histone marks (e.g., H4K20me2/3, H3K9me) are not affected and produce a cell cycle arrest phenotype, similar to that of SETD8 knockdown. In addition to the lack of selectivity against other protein methyltransferases, a few off-target effects on other cellular targets (inhibition of Cdc25 for SPS8I1; inhibition of cyclin-dependent kinases 4 and 2 (CDK4/2) for SPS8I2; and inhibition of protein tyrosine phosphatase PTB1B for SPS8I3) have been observed and documented [97]. More recently, the same authors also developed and patented a series of naphthoquinone derivatives, the more active of them (namely compounds SGSS05-N, SGSS05-NS, and SPECS-21) inhibit SETD8 with IC_50_ values ≤5 μM. Again, the compounds also inhibit other KMTs (SETD2, SETDB1, GLP, G9a, SMYD2, SMYD3, MLL1 and SETD7) and PRMTs (PRMT1, PRMT3, CARM1, PRMT8) in the low micromolar range [98].

## Conclusions

An increasing number of reports on the key role played by lysine methyltransferase SETD8 in physiological and pathological pathways have appeared in literature during the last decade. Many questions are still unsolved and the effects of the enzyme in both normal and diseased cells are still far from being completely understood, yet, as described in this review, it is evident that SETD8 contributes to the regulation of transcriptional activity and that it is implicated in many human diseases comprising cancer. However, regardless tremendous progress in the discovery of selective, small-molecule inhibitors of protein methyltransferases, only a limited number of inhibitors have been reported so far for SETD8, with just a few of them being endowed with a certain degree of selectivity and/or cellular activity. Moreover, the structures of most of the inhibitors identified so far (if not all of them) contain Pan Assay INterference compounds (PAINS) [99, 100] motifs and, therefore, could exhibit multiple behaviors that could interfere in assay readouts, such as metal chelation, redox cycling, and protein reactivity [101]. The improvement of these lead compounds as well as the discovery of novel molecular scaffolds will hopefully help the investigation of the biology of this emerging target and could lead to the development of novel therapeutics. In this regard, the recent identification of the domain used by SETD8 to bind and methylate the nucleosome [24] could open the way to design a new generation of allosteric inhibitors.
